# Cytotoxic effects of ergone, a compound isolated from *Fulviformes fastuosus*

**DOI:** 10.1186/s12906-016-1471-8

**Published:** 2016-11-25

**Authors:** Dilusha Fernando, Achyut Adhikari, Chandrika Nanayakkara, E Dilip de Silva, Ravi Wijesundera, Preethi Soysa

**Affiliations:** 1 Department of Plant Sciences, Faculty of Science, University of Colombo, Colombo, 03 Sri Lanka; 2 Department of Molecular Biology and Biochemistry, Faculty of Medicine, University of Colombo, Colombo, 03 Sri Lanka; 3 Department of Chemistry, Faculty of Science, University of Colombo, Colombo, 03 Sri Lanka; 4 The Hussain Ebrahim Jamal Research Institute of Chemistry, ICCBS, University of Karachi, Karachi, Pakistan

**Keywords:** Mushrooms, *Fulviformes fastuosus*, Isolation method, Ergone, Cytotoxic activity, Rhabdomyosarcoma, Hepatocellular carcinoma

## Abstract

**Background:**

Mushrooms inspired the cuisines of many cultures and conventional medicaments for cancer. However, a substantial number of mushroom species are yet unexplored, possessing an unknown chemical, biological and pharmacological profiles. *Fulviformes fastuosus* is a terrestrial mushroom, which is commonly found in Sri Lankan woodlands. The current study was aimed at isolation and characterization of a potent cytotoxic compound from *F. fastuosus* and investigating the apoptotic effect induced by the active principle against cancer and normal cell lines.

**Methods:**

Bioactivity guided isolation of active principles from the methanol extract of *F. fastuosus* was performed by a rapid extraction and isolation method using different chromatographic techniques. Potential cytotoxic compound was identified using one and two dimensional nuclear magnetic resonance spectroscopy and mass spectrometry. Isolated compound was screened for in vitro cytotoxicity against Hepatocellular carcinoma (HepG-2), Muscle rhabdomyosarcoma (RD) and Rat Wistar liver normal (CC-1) cell lines using 3 4, 5-(dimethylthiazol-2-yl) 2-5-diphenyl tetrazolium bromide (MTT) cell viability assay. Apoptotic features of cells were observed via microscopic examination and ethidium bromide/acridine orange fluorescent staining.

**Results:**

The interpretation of spectral data resulted in the identification of the chemical structure as ergosta-4,6,8 (14),22-tetraen-3-one (ergone). Ergone exhibited promising cytotoxic properties against RD cells with less cytotoxicity effect on CC-1 cells. In addition, ergone also possesses a strong cytotoxic effect against HepG-2 cells showing low toxic level for CC-1 cells. Apoptotic features of treated cells were detected via morphological characterization and ethidium bromide/acridine orange staining.

**Conclusion:**

The present study elaborates the isolation of a potent cytotoxic compound; ergone, from F. fastuosus via a rapid and efficient isolation method. Importantly, ergone has exhibited greater cytotoxic activity against RD cells with high selectivity index compared to cytotoxicity against HepG-2 cells. Ergone can be used in the development of therapeutic strategies for curbing rhabdomyosarcoma.

**Electronic supplementary material:**

The online version of this article (doi:10.1186/s12906-016-1471-8) contains supplementary material, which is available to authorized users.

## Background

Mushrooms have long been esteemed as edible and medicinal provisions for humankind. From ancient time, substances derived from mushrooms are considered as a boundless source of anticancer agents [1, 2]. Among the prevailing degenerative diseases in the globe, cancer has become the most prevalent devastating human degenerative disease worldwide [3, 4]. In spite of the availability of modern antineoplastic agents, the number of lives taken by this malicious disease increases annually around the globe. The recent advances in cancer therapies have failed in making significant progress in reducing the morbidity caused by cancer [5, 6]. In this perspective, the use of secondary metabolites derived from natural sources having promising cytotoxic properties in the development of new and more effective anticancer drugs are highly desirable.

Mushrooms provide potent beneficial effects on cancer either directly as antioxidants or through prevention of genetic alterations underlying cancer [7]. The secondary metabolites derived from mushrooms have the potential to play a leading role in the development of anticancer drugs with minimal side effects [8, 9]. There are approximately 650 species of macrofungi that have been reported to possess antitumor activity [10]. Calvacin was the most commonly used anticancer compound isolated from giant puffball mushrooms [11]. In addition, lentinan, schizophyllan and krestin derived from mushrooms are approved as prescription drugs for the treatment of cancer in Japan [12]. Currently, fungal extracts and isolated fungal fractions are also being used in the traditional medicine for the treatment of cancer as adjuvants to chemotherapy, surgery and radiotherapy [13, 14].

Despite the development of anticancer therapeutics using mushrooms, a large proportion of mushroom species in the world still remain scientifically unexplored making this group one of the mostly untapped microbiotic source for novel drug leads in the anticancer pharmaceutical industry [15]. Intriguingly, the mycological potential of tropical island Sri Lanka is attributable to favorable climatic conditions and floral diversity [16, 17]. However, ethnomycological research in Sri Lanka indicates only a rare use of mushrooms as food or internal medicinal provisions by indigenous practitioners in Sri Lanka [18, 19]. Hence, the current study was aimed at isolation and characterization of a potent cytotoxic compound from *Fulviformes fastuosus* harvested from the dry zone forest reserves in Sri Lanka. *F. fastuosus* belongs to the Hymenochaetaceae family, which is commonly found in dry zone woodlands in Sri Lanka. Members of the Hymenochaetaceae family are known to possess strong bioactive properties including antioxidant and anticancer activity which is strongly attributed to polyphenolic substances contained in the mushrooms [20]. Since the polyphenolic secondary metabolites produced by *F. fastuosus* mushroom exhibit largely diverse structural differences, the isolation and separation of metabolites can be lengthy and tedious [21]. Therefore, the methods of isolating bioactive compounds are generally optimized to a series of rapid, simple and efficient bioactivity guided fractionations [22, 23].

## Methods

### Chemicals and equipment

Methanol, HCl, hexane, chloroform and ethyl acetate were purchased from BDH Chemicals (Poole, England). MTT (3-(4,5-Dimethylthiazol-2-Yl)-2,5-Diphenyltetrazolium Bromide), Ethidium bromide, Cycloheximide, Isoamyl alcohol, and Acridine orange were purchased from Sigma Chemicals Co. (St. Louis, USA). All chemicals used were of analytical grade.

Shimadzu UV 1601 UV visible spectrophotometer (Shimadzu Corporation, Kyoto, Japan) was used to measure the absorbance. Rotary evaporator (BUCHI Rota vapor R-200) was used to dry the extracts of *F. fastuosus*. Thin layer chromatography was carried out by using pre coated silica gel 60 F _254_ (20×20) cm^2^ Aluminum backed commercial grade sheets. Chromatograms were visualized using UV radiator with 9815 series lamp (254–364 lambda). Nuclear magnetic resonance (NMR) spectra were recorded on a Bruker avance AV- 600 MHz spectrometer with a 5 mm cryoprobe.

Cells were incubated at 37 °C in a humidified CO_2_ incubator (SHEL LAB/Sheldon manufacturing Inc. Cornelius, OR 97113, USA). Olympus (1X70-S1F2) inverted fluorescence microscope (Olympus Optical Co. Ltd. Japan) was used for the observation of cells and images were captured using digital camera (MDC 200, USB 2.0, 2 M pixels CCD chip) connected to the microscope. Deionized water from LABCONCO (waterproplus) UV ultra-filtered water system (LABCONCO Corporation, Kansas city, Missouri 64132–2696) or distilled water was used in all experiments.

### Fungal material

The specimen of *F. fastuosus* was collected from the dry zone forest reserves of Dambulla, Sigiriya, and Minneriya in Sri Lanka and transported to the laboratory with proper ventilation. The identity of the specimen was authenticated by a botanist of Department of Plant Science, Faculty of Science, University of Colombo, and molecular studies confirmed the identity of the species (Genbank Accession No.: KP757737). Voucher specimens were deposited at the same Institute (UOC:DAMIA: D27b).

### Large scale extraction of fruiting bodies of *F. fastuosus*

Mature fruiting bodies of *F. fastuosus* were brush cleaned, dried in the oven at 40 °C to a constant mass and pulverized. Shredded and ground mushroom materials of fruiting bodies from *F. fastuosus* (1 kg) were subjected to sonication extraction with methanol (4 L), for 5–6 h at room temperature. Methanol extract was filtered twice through Whatman No. 1 filter paper and same extraction procedure was repeated for the residue. Filtrates were combined and evaporated to dryness at 40 °C under reduced pressure using rotary evaporator to remove methanol. The resulting dried methanol extract was dissolved in distilled water (500 mL) and was partitioned in to hexane, dichloromethane and ethyl acetate (500 mL each), respectively. Ethyl acetate fraction of *F. fastuosus* was evaporated in a rotary evaporator to be used in subsequent fractionation and isolation of bioactive components.

## Fractionation, isolation and structure elucidation of the bioactive component

### Bioactivity guided isolation of active principles using chromatographic techniques

The dried ethyl acetate fraction of *F. fastuosus* was dissolved in a minimum amount of methanol and mixed with 5 g of silica gel (230–400 mesh, 60 Å). The resulting silica gel slurry was dried using rotary evaporator and placed at the top of 10 mm diameter column filled with silica gel to produce final bed height of 100 mm. The column of silica was eluted, using a gradient solvent system starting from 100% hexane to 1:1 hexane: ethyl acetate (100 mL each in 5% step gradient). Based on the bioactivity and TLC studies, the eluted fraction numbers 30–34 using 70: 30 hexane: ethyl acetate solvent system were preferred for further isolation and purification of active compound.

The combined fraction numbers 30–34 were concentrated to dryness at reduced pressure. Resulting residue was dissolved in methanol and mixed with silica gel as mentioned previously. Subsequently, the gel slurry was introduced to a similar size (10 mm diameter; final bed height of 100 mm) of normal phase silica column packed with silica. The column of silica was eluted using a gradient solvent system starting from 100% hexane to 75:25 hexane: ethyl acetate solvent system (1% step gradient). Based on the bioactivity and TLC studies, the eluted fractions number 21–25 using 91: 9 hexane: ethyl acetate solvent system were subjected to preparative thin-layer chromatography (developed twice with hexane/ethyl acetate, 9:1). Subsequently, it resulted in 15 mg of a white solid, which proved to be nearly pure by TLC analysis where compound was dissolved in CH_3_OH and spotted on normal phase TLC plates using pet. ether/ethyl acetate (3:l) mixture to give a consolidated under 254 nm UV lamp. Final purification of the compound was achieved by recrystallization from methanol.

### Identification and characterization of the compounds by nuclear magnetic resonance (NMR) and mass spectrometry (MS)

One and two dimensional NMR spectra, including ^1^H NMR, ^13^C NMR, DEPT ^13^C NMR, COSY, HMQC, HMBC and NOESY were recorded to obtain chemical shift information of the active compound using CD_3_OD as the solvent. Electrospray Ionization Mass Spectrometry (EIMS) was performed to obtain the high resolution mass spectrum.

### Cell lines and cell culture

The RD, HepG-2 and CC-1 cell lines were used in the determination of in vitro cytotoxicity of isolated compound. Human muscle rhabdomyosarcoma (RD) and Rat liver normal (CC-1) cell lines were obtained from Medical Research Institute, Colombo 08. Human hepato cellular carcinoma (HepG-2) cell line was obtained from Dr. Panjwani Center for Molecular Medicine and Drug Research, International Center for Chemical and Biological Sciences, University of Karachi. The cells were cultured and preserved in the Department of Biochemistry and Molecular Biology until used them in cytotoxicity experiments. The cells were cultured in DMEM supplemented with 10% heat inactivated fetal bovine serum (FBS), HEPES, 3% glutamine, sodium bicarbonate and antibiotic (penicillin/streptomycin). All the cultured cell lines were incubated at 37 °C in a humidified CO_2_ incubator.

## In vitro cytotoxicity and identification of the apoptotic features of treated cells with isolated compound from *F. fastuosus*

### MTT assay

The RD and Hep-G2 cells were seeded in 24-well plates at the density of 2 × 10^5^ cells per well and CC-1 cells were cultured at a density of 2 × 10^3^ cells per well followed by incubation at 37 °C for overnight in a humidified CO_2_ incubator. Test compound was dissolved in methanol: DMSO (1:1) mixture and confluent monolayers were treated with different concentrations of the above prepared test solution followed by incubation at 37 °C for 24 h. In all experiments, cycloheximide (5 mM, 50 μL) was used as the positive control and negative control contained the growth media and the solvent mixture used to dissolve the test compound. Subsequently, the cells treated with test solution were subjected to MTT cell viability assay. The culture medium was replaced with fresh growth medium (1 mL) and MTT (5 mg/mL; 100 μL) was added to each well. The cells were incubated at 37 °C for 3 h and the growth medium was aspirated carefully. The remaining formazan crystals were solubilized in 750 μL of 0.05 M HCl prepared in 2-propanol and absorbance was measured at 570 nm. Percentage cell viability was determined using the formula: Percentage cell viability = [(Absorbance of untreated cells -Absorbance of treated cells)/Absorbance of untreated cells]*100 (Additional file 1). The net absorbance from the wells of the negative control was considered as the 100% viability. EC_50_ values were determined by regression analysis (*R* ^*2*^ > 0.95) of the corresponding dose response curves of percentage inhibition of cell viability and concentration of the test solution.

### Morphological determination

The apoptotic morphological changes of treated cells with different concentrations of the test solution over 24 h were observed via microscopic examination of cells. Morphological changes were compared with negative and positive controls under the light microscope.

### Acridine orange-Ethidium bromide (AO/EB) staining

Apoptotic morphology of treated cells was detected and distinguished by acridine orange-ethidium bromide (AO/EB) fluorescent staining. The staining method was performed via introducing a mixture of fluorescent dyes, acridine orange and ethidium bromide in 1:1 ratio on treated cells. RD, HepG-2 cancer cells (2 × 10^3^ cells/well) and CC-1 cells (2 × 10^2^ cells/well) were seeded in chamber slides. Resulting confluent layers were treated with different concentrations of the test solution and incubated at 37 °C for 24 h. The adherent cells were washed with 200 μL of PBS and 2 μL of the dye mixture containing ethidium bromide (100 mg/mL) and acridine orange (100 mg/mL) in 1:1 ratio was placed on each well of the chamber slide. Chamber slides were examined immediately under the florescence microscope and images were captured.

### Calculations and statistics

All experiments were performed in triplicate and values given are representative for at least three independent experiments. All the results of the experiments were expressed as the mean ± standard deviation (Mean ± SD). The results were statistically analyzed by *T* test using Microsoft Excel. A *p*-value less than 0.05 were considered as statistically significant.

### Ethical approval

The requirement for ethical approval was waived.

## Result

### Identification of compound isolated from the extract of *F. fastuosus*

**Fig. 1:**
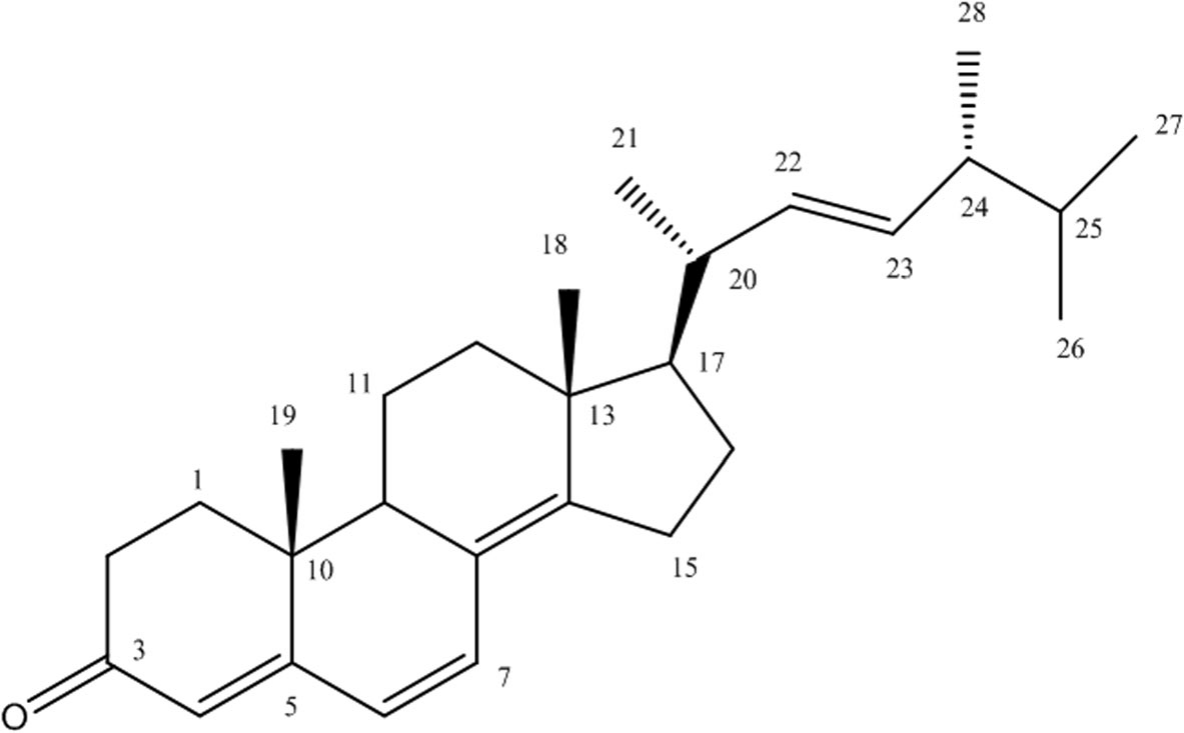
Structural Formula of Ergone

Purified compound was obtained from the extract of *Fulviformes fastuosus* to yield 4 mg of the compound as white colour needle shaped crystals. The EIMS showed molecular ion peak at *m/z* 392. Its molecular formula, C_28_H_40_O was determined by HREI MS which showed molecular ion peak *m/z* at 392.3090 (calcd for C_28_H_40_O = 392.3079). The ^1^H-NMR spectrum (Table 1) displayed signals for six methyl groups at δ 0.83 (3H, d, *J* = 7.5 Hz, H_3_-26), 0.85 (3H, d, *J* = 7.5 Hz, H_3_-26), 1.03 (3H, s, H_3_-18), 1.06 (3H, s, H_3_-19), 1.07 (3H, d, *J* = 7.5 Hz, H_3_-21), 1.08 (3H, d, *J* = 7.5 Hz, H_3_-28). Additionally, the ^1^H-NMR spectrum displayed resonances for five downfield signals at δ 5.26 m (H-22), 5.27 m (H-23), 5.70 s (H-4), 6.10 d (*J* = 9.6 Hz, H-6), 6.71 d (*J* = 9.6 Hz, H-7). The structure of compound was further confirmed by 2D-NMR spectra (COSY, HSQC, HMBC, NOESY) (Additional file 2). The spectral data were in good agreement with those of ergosta-4,6,8 (14),22-tetraen-3-one which was previously reported by Youla et al.*,* (1992) [24]. Hence, the interpretation of above spectral data (Table 1) resulted in the identification of the chemical structure of purified compound as ergosta-4,6,8 (14),22-tetraen-3-one or ergone (Fig. 1).

**Table 1:** ^13^C-and ^1^H-NMR chemical shift values of ergone (ppm, CD_3_OD, 150 and 600 MHz, respectively)

C. No.	δ_C_	δ_H_ (*J*, Hz)
1	35.2	–
2	34.8	–
3	202.3	–
4	123.2	5.70 s
5	167.9	–
6	125.3	6.10 d (9.6)
7	136.7	6.71 d (9.6)
8	125.9	–
9	45.7	–
10	38.0	–
11	20.0	–
12	36.8	–
13	45.2	–
14	157.5	–
15	26.2	–
16	27.6	–
17	57.2	1.29 m
18	16.9	1.03
19	18.2	1.06
20	40.6	–
21	20.1	1.07
22	133.7	5.26 m
23	130.8	5.27 m
24	44.4	–
25	35.4	2.12
26	20.5	0.83 d (7.5)
27	21.7	0.85 d (7.5)
28	20.4	1.08 d (6.4)

### In vitro Cytotoxicity of ergone

**Fig. 2:**
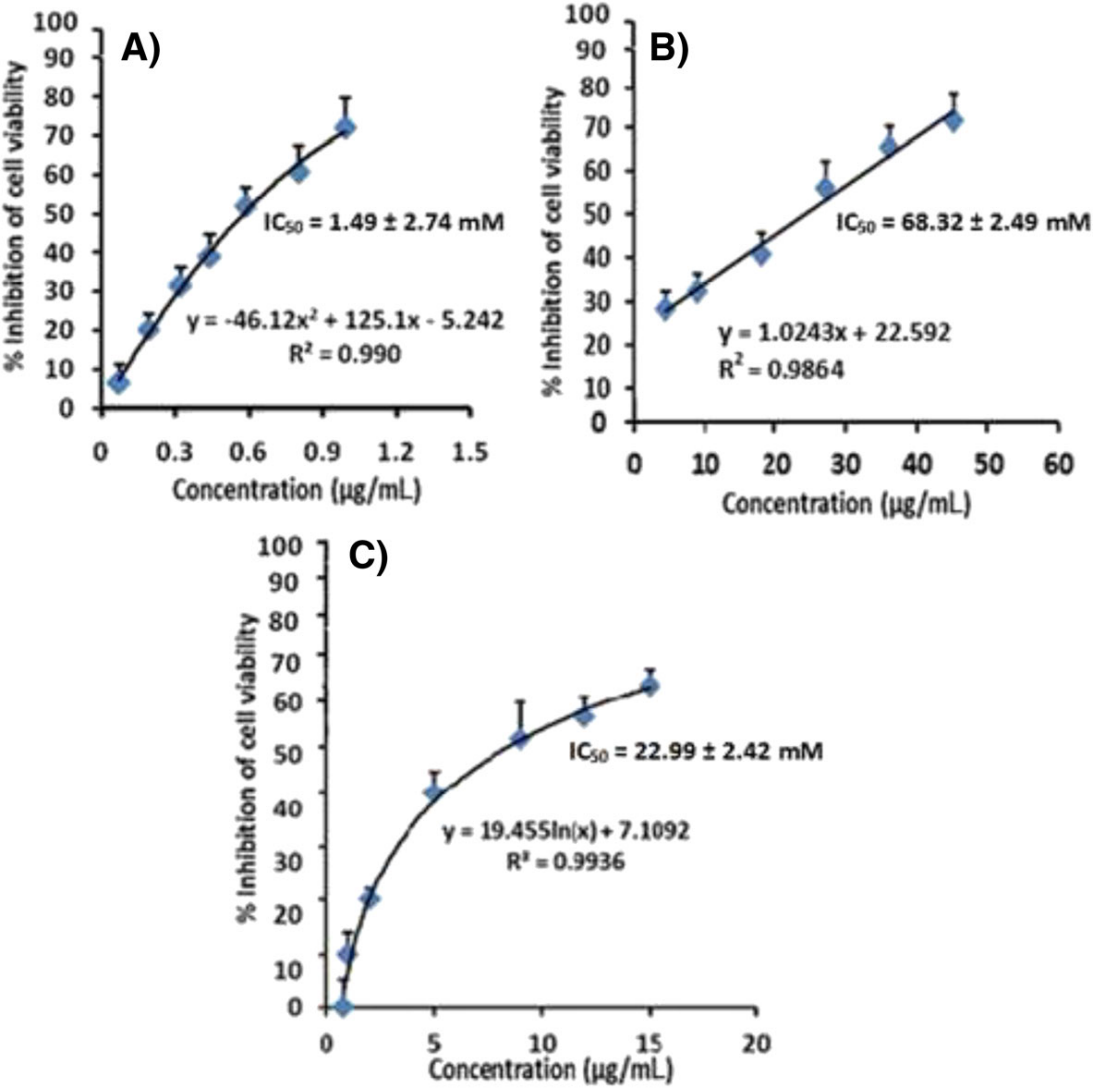
The percentage inhibition of cell viability against (**a**) RD (**b**) Hep-G2 (**c**) CC-1 cell line as determined by MTT assay, after 24 h treatment with the ergone. The graphical data are represented as mean ± SD of three independent experiments (*n* = 3)

As evident in Fig. 2a, ergone demonstrated a potent cytotoxic effect on RD cells with remarkably lower IC_50_ value of 1.49 ± 2.74 mM. Moreover, Fig. 2a depicts a dose dependent increase of percentage inhibition of cell viability over a concentration range of 0.01–1.5 μg/mL of test solution. The maximum of 74% inhibition of cell growth was observed at 5.0 μg/mL of the test solution. Ergone also exhibited a strong cytotoxicity effect against HepG-2 cells by showing an IC_50_ of 68.32 ± 2.49 mM in a dose dependent manner (Fig. 2b). Positive control (cycloheximide) exhibited 76.35 ± 3.12% growth inhibition at the concentration (5 mM, 50 μL) used.

The IC_50_ value determined for the mean of the three independent sample preparations of ergone dissolved in methanol: DMSO (1:1) against normal mammalian cell line (CC-1) was 22.99 ± 2.42 mM (Fig. 2c). Thereby, selectivity index shown by the compound ergone against RD cells becomes 15.44 indicating less cytotoxic effect on normal cells according to the definition of Prayong et al.*,* (2008) [25].

### Morphological changes

**Fig. 3:**
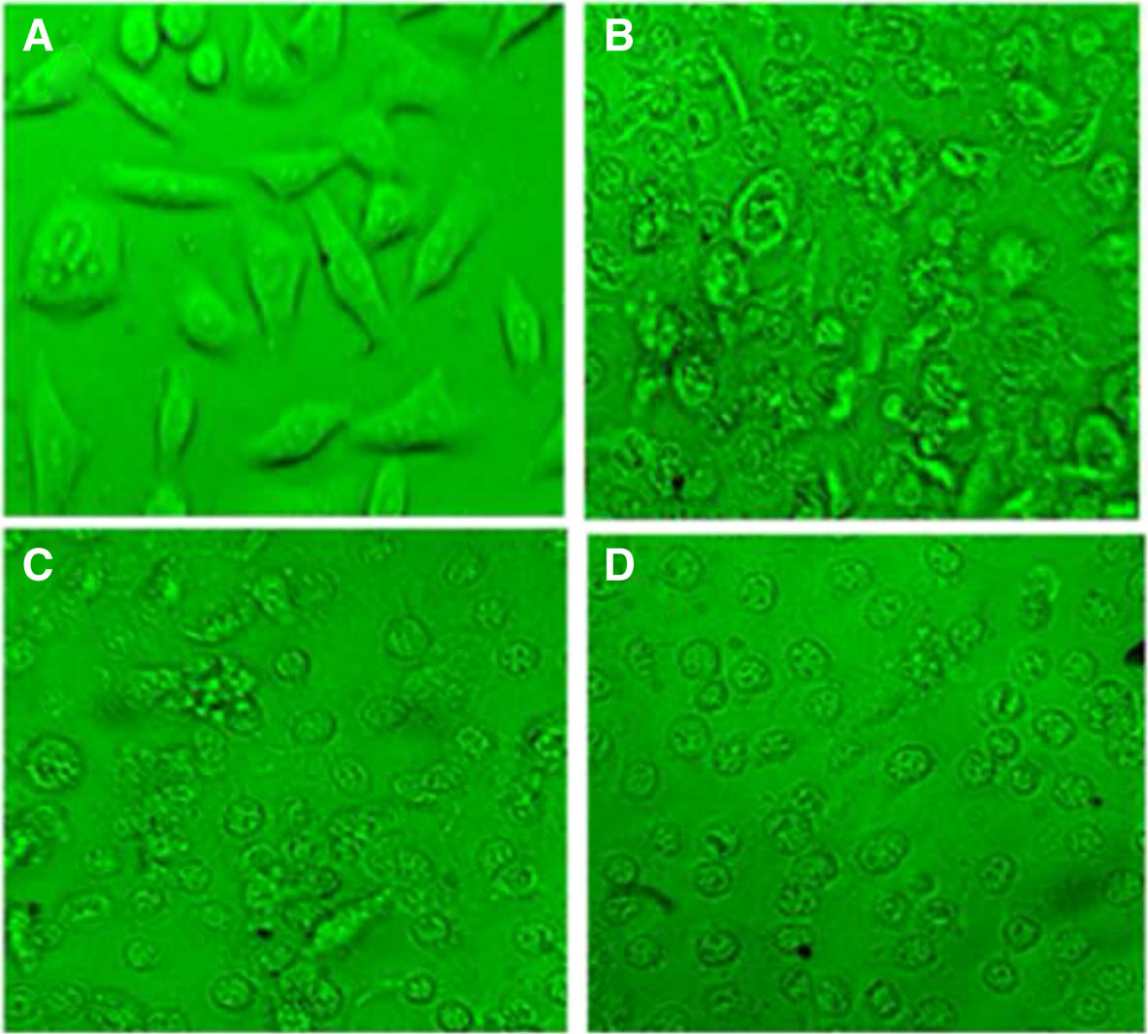
Light micrographs of RD cell line after 24 h of incubation with ergone at different concentrations. **a**- 0.1 μM; (**b**)- 2 μM; (**c**)- 50 μM; (**d**)- Cycloheximide as the positive control (5 mM; 50 μL) (×40)

**Fig. 4:**
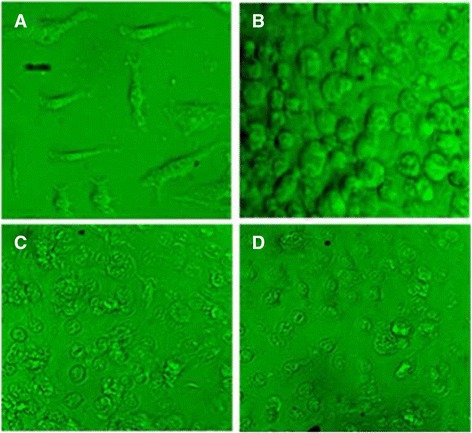
Light micrographs of Hep-G2 cell line after 24 h of incubation with ergone at different concentrations. **a**- 5 mM; (**b**)- 50 mM; (**c**)- 80 mM; (**d**)- Cycloheximide as the positive control (5 mM; 50 μL) (×40)

**Fig. 5:**
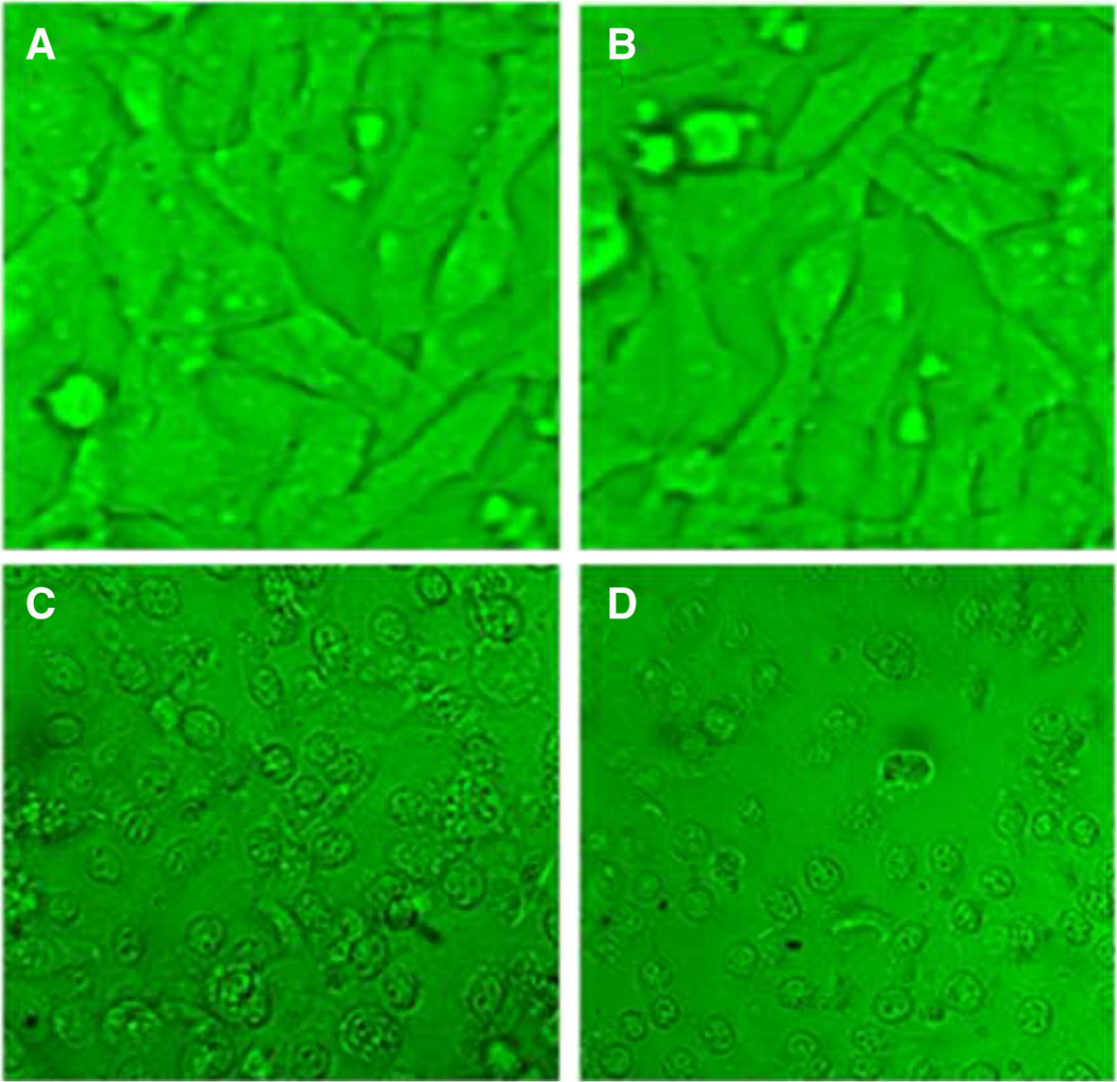
Light micrographs of CC-1 cell line after 24 h of incubation with ergone at different concentrations. **a**- 2 mM; (**b**)- 10 mM; (**c**)- 40 mM; (**d**)- Cycloheximide as the positive control (5 mM; 50 μL) (×40)

The morphological changes observed on RD, HepG-2, and CC-1 cells after the treatment with ergone are represented in Figs. 3, 4 and 5. The morphology of untreated cells and cells treated with the lower concentrations of ergone appeared in elongated shape. The cells treated with higher doses exhibited apoptotic features including cellular shrinkage, oval or irregular shape, fragmented nuclei, apoptotic bodies formation and condensed cytoplasm. The concentration of ergone in which cells started to display apoptotic features varied depending on the type of the cell line.

### Ethidium bromide/acridine orange staining

**Fig. 6:**
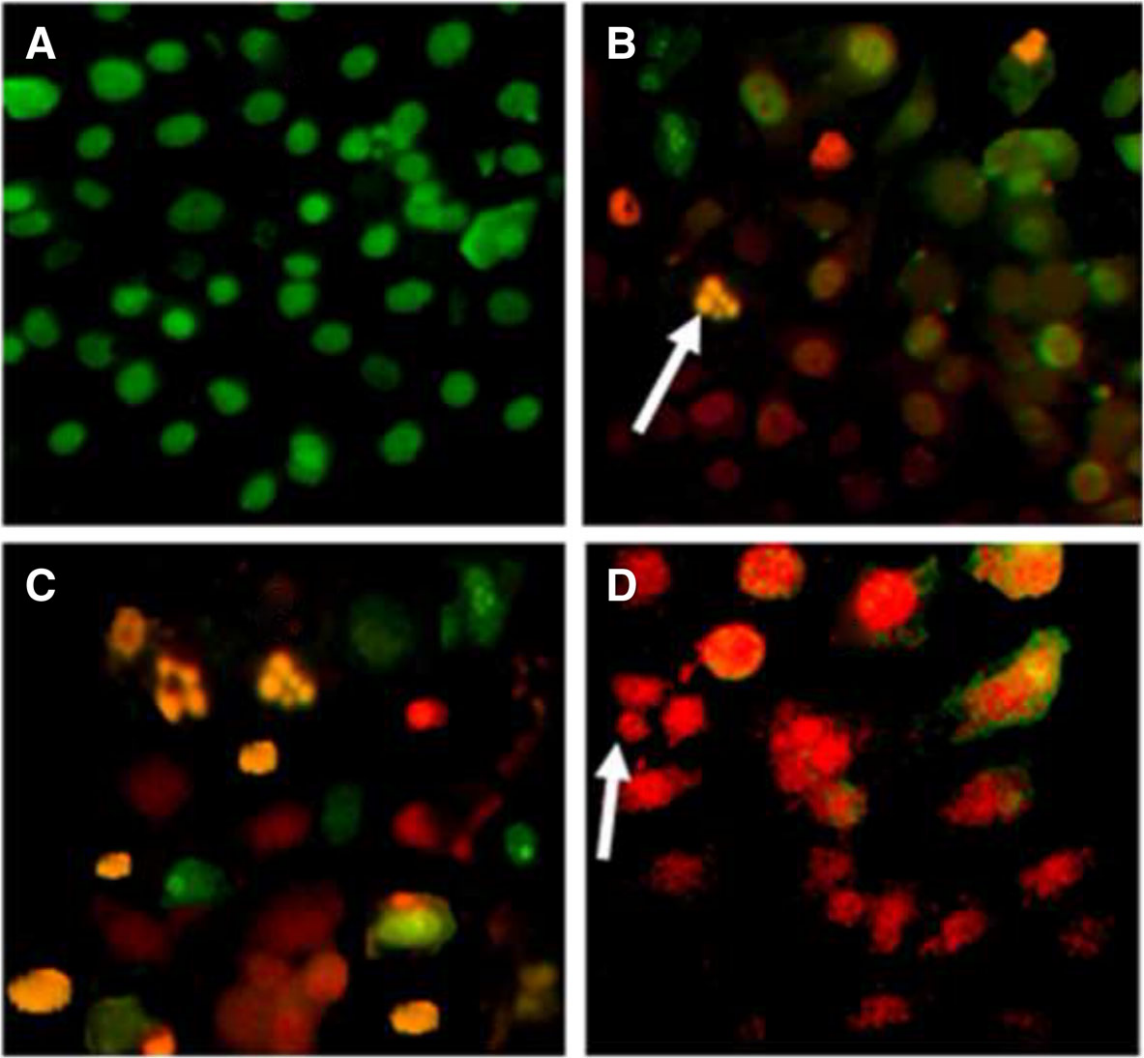
Apoptotic morphology detection by acridine orange-ethidium bromide (AO/EB) fluorescent staining of RD cell line treated with ergone dissolved in methanol: DMSO (1:1). **a**- Negative control; (**b**)- 2 μM; (**c**)- 5 μM; (**d**)- Cycloheximide as the positive control (5 mM; 50 μL) (×40). *Arrows* indicate formation of apoptotic bodies

**Fig. 7:**
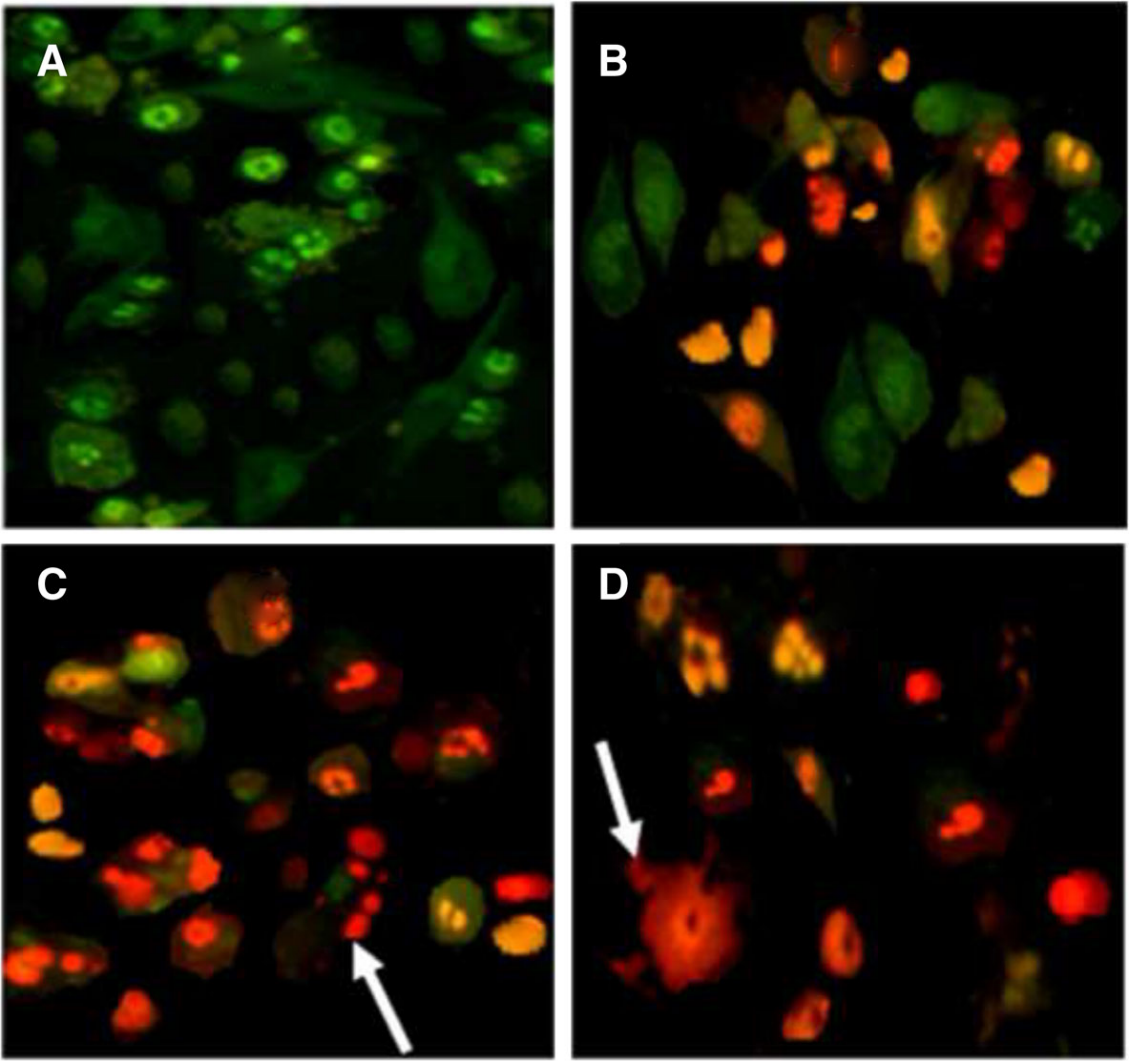
Apoptotic morphology detection by acridine orange-ethidium bromide (AO/EB) fluorescent staining of HepG-2 cell line treated with ergone dissolved in Methanol: DMSO (1:1). **a**- Negative control; (**b**)- 80 μM; (**c**)- 130 μM; (**d**)- Cycloheximide as the positive control (5 mM; 50 μL) (×40). *Arrows* indicate formation of apoptotic bodies

**Fig. 8:**
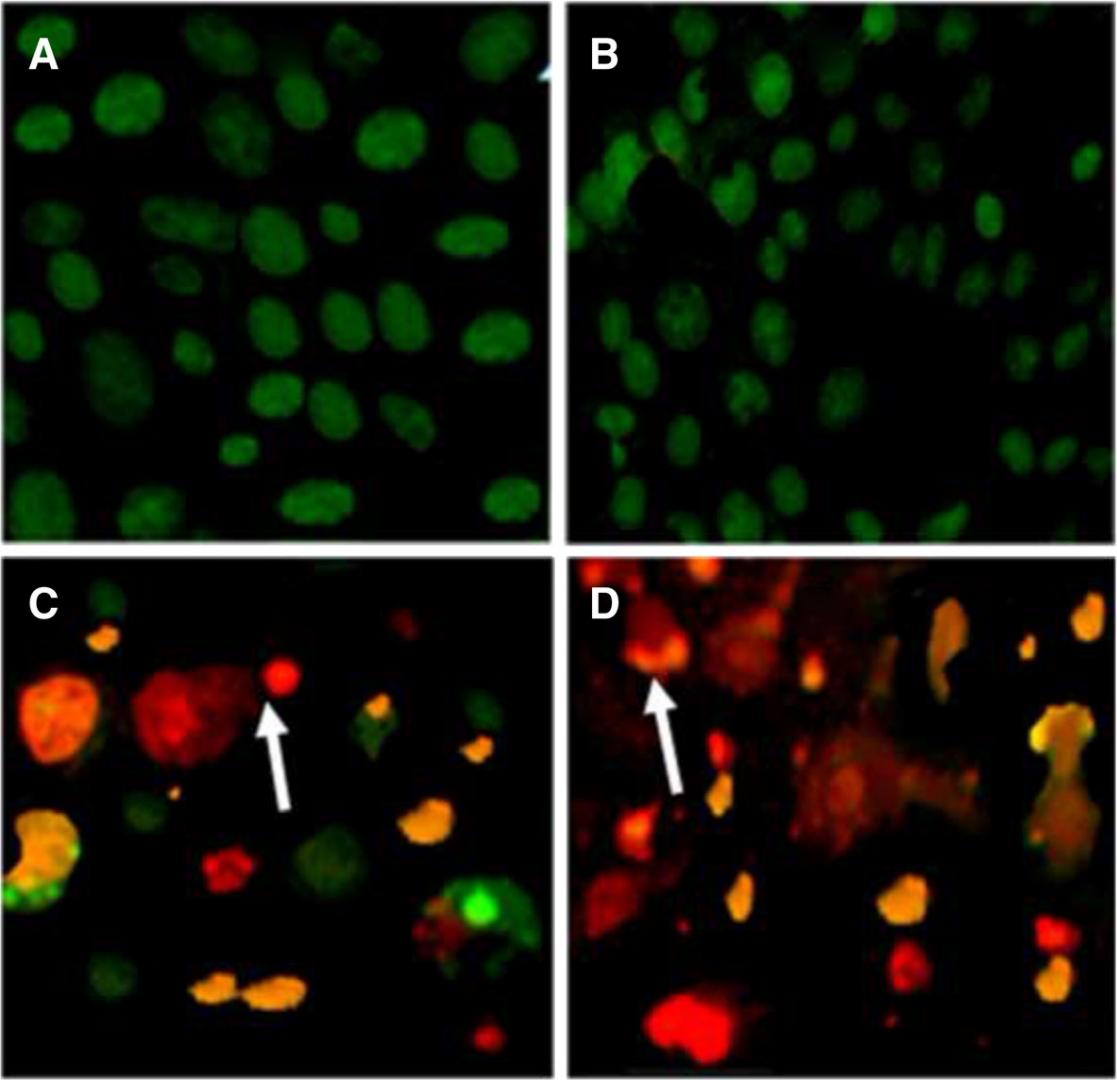
Apoptotic morphology detection by acridine orange-ethidium bromide (AO/EB) fluorescent staining of CC-1 cell line treated with ergone dissolved in Methanol: DMSO (1:1). **a**- Negative control; (**b**)- 6 μM; (**c**)- 50 μM; (**d**)- Cycloheximide as the positive control (5 mM; 50 μL). This figure denotes the results of at least 3 independent experiments (Original magnification 40×). *Arrows* indicate formation of apoptotic bodies

As evident in Figs. 6, 7 and 8, the apoptotic morphology was detected by acridine orange-ethidium bromide (AO/EB) fluorescent staining of RD, HepG-2 and CC-1 cell lines treated with ergone.

As demonstrated in Figs. 6, 7 and 8, nuclei stained with green colour demonstrate live cells while greenish yellow shows early apoptotic cells. Condensed Orange red nuclei indicate late apoptotic cells whereas red colour exhibits dead cells. The untreated cells (negative control) and treated cells with the lower concentrations of the compounds appeared with the normal nuclei presented in bright green. Cells treated with higher doses of the compounds and 5 mM concentration of positive control showed unique features of apoptosis such as chromatin condensation, nuclear fragmentation, presence of apoptotic bodies and blebbing formation, are of significance upon examination of stained cells with ethidium bromide/acridine orange. Chromatin condensation and nuclear fragmentation were predominantly observed apoptotic features for treated cells with high concentrations of the compounds. In addition, number of apoptotic cells was gradually increased with the treatment dose.

## Discussion

The past decade has witnessed the overwhelming interest in isolation and characterization of bioactive compounds from medicinal mushrooms due to the fact that natural products of mushroom origin are found to possess a remarkably high pharmacological index [26, 27]. The mounting evidences from various scientific studies regarding the applications of mushroom extracts and substances in the development of anti-cancer therapeutics unarguably make it a fast-track research area worth mass attention. Consequently, mushroom consumption has been shown to prevent the development of cancer later in life with studies reporting an inverse correlation between mushroom intake and the risk of developing cancer [28, 29]. In this perspective, the current study has been succeeded in the isolation of a potent cytotoxic compound namely ergone which is derived from a Sri Lankan mushroom. Ergone is an essential steroid, which belongs to ergosterol group [30]. Since it is necessary to expand the current extraction protocols with the increasing interest in mushroom metabolites, a novel and easy isolation method have been optimized in the current invention to isolate ergone with high reproducibility*.* Specifically, the extraction and isolation process of ergone is rapid, simple, inexpensive and comprehensive with respect to the constituents to be isolated.

In vitro cytotoxic activity of isolated product ergone against RD and HepG-2 cells were determined by MTT cell viability assay, which is a well-established colorimetric method to assess the cell proliferation and cell viability. It has been revealed that ergone isolated from *F. fastuosus* has promising cytotoxic properties against RD cells with less cytotoxicity effect on normal CC-1 cells (SI = 15.44). Moreover, ergone also showed strong cytotoxic activity against HepG-2 cells being less toxic to normal CC-1 cells. However, cytotoxic activity of ergone against RD cell line is significantly high compared to HepG-2 cells (*p* < 0.05). The apoptotic features observed via microscopic examination of treated cells and AO/EB fluorescent staining evidenced the apoptosis induced by ergone against RD and HepG-2 cells. Morphological characterization and ethidium bromide/acridine orange staining of treated cells revealed that the cell death induced by ergone has mediated through apoptosis. It strengthens the results obtained via MTT cell viability assay.

Our previous study has revealed that the crude methanolic extract of *F. Fastuosus* possess cytotoxic activity against RD cell line with an IC_50_ of 14.72 ± 1.56 μg/mL [31]. Ergone’s cytotoxic activity against RD cell line (584.01 ± 2.74 ng/mL) has become twenty five times greater than the cytotoxic activity shown by the crude extract of *F. fastuosus* in terms of IC_50_ against RD cell line. Hence, it can be concluded that the promising cytotoxic activity of *F. fastuosus* could be largely contributed by the remarkably high cytotoxic activity of ergone. In addition, mangiferin, a strong anticancer compound isolated from *Mangifera indica* has shown an inhibitory concentration (IC_50_) of 33.40 μg/mL against RD cells [32]. Interestingly, antiproliferative activity shown by ergone was found to be significantly greater (*p* < 0.05) than the anticancer activity of mangiferin. Importantly, the present discovery has become the first study of isolating ergone from *F. fastuosus* and it is appeared to be an essential anti-tumor drug lead against rhabdomyosarcoma. Currently, the treatment regime of rhabdomyosarcoma comprises of chemotherapy and radiation along with surgery while producing adverse side effects including neurotoxicity, hepatotoxicity and cardiotoxicity [33]. Hence, the use of afore kind of natural products against RD is an extremely promising strategy for remedy of rhabdomyosarcoma. Intriguingly, most of the mushrooms belong to the family of Hymenochaetaceae have long been used as edible forms [34]. Therefore, ergone, which is isolated from family Hymenochaetaceae, has a greater potential to act safely in human body without creating any complications. In addition, this fact was supported by the high selectivity index shown by the ergone against RD sarcoma cells.

## Conclusion

Ergone is a potent cytotoxic compound against RD and HepG-2 cancer cell lines being less toxic to normal CC-1 cells. Moreover, ergone has shown significantly greater cytotoxic activity against RD cell line compared to HepG-2 cell line. The present findings imply the propensity of ergone to develop as a safe pharmaceutical composition against rhabdomyosarcoma with a high efficacy for inducing cell death of RD while being less toxic to normal cells. Intriguingly, above discovery provides a scientific proof of the traditional awareness in using natural compounds isolated from medicinal mushrooms as anticancer agents.

## Additional files

Additional file 1: A summary of the data obtained from MTT assay. (XLSX 10 kb)
Additional file 2: 1D, 2D and mass spectra. (DOCX 12600 kb)

## Abbreviations

^13^ C NMR: Carbon nuclear magnetic resonance spectroscopy
^1^H NMR: Proton nuclear magnetic resonance spectroscopy
AO/EB: Acridine orange (AO)/ethidium bromide (EB)
CD_3_OD: Deuterated methanol
CH_3_OH: Methanol
COSY: H-H correlation spectroscopy
DEPT ^13^C NMR: Distortionless enhancement polarization transfer carbon nuclear magnetic resonance spectroscopy
DMSO: Dimethyl sulfoxide
EIMS: Electrospray ionization mass spectrometry
HCl: Hydrochloric acid
HMBC: Heteronuclear multiple-bond correlation spectroscopy
HMQC: Heteronuclear multiple-quantum correlation spectroscopy
MTT: 3 4, 5-(dimethylthiazol-2-yl) 2-5-diphenyl tetrazolium bromide
NMR: Nuclear magnetic resonance
NOESY: Nuclear Overhauser effect spectroscopy
SI: Selectivity index
TLC: Thin layer chromatography
