# Supplementary material for: Cytotoxic effects of ergone, a compound isolated from Fulviformes fastuosus
Source: BMC Complement Altern Med. 2016 Nov 25;16:484. doi: 10.1186/s12906-016-1471-8 (PMC5124230; doi:10.1186/s12906-016-1471-8)
Supplement: Additional file 2: — 1D, 2D and mass spectra. (DOCX 12600 kb) [file 12906_2016_1471_MOESM2_ESM.docx]

**
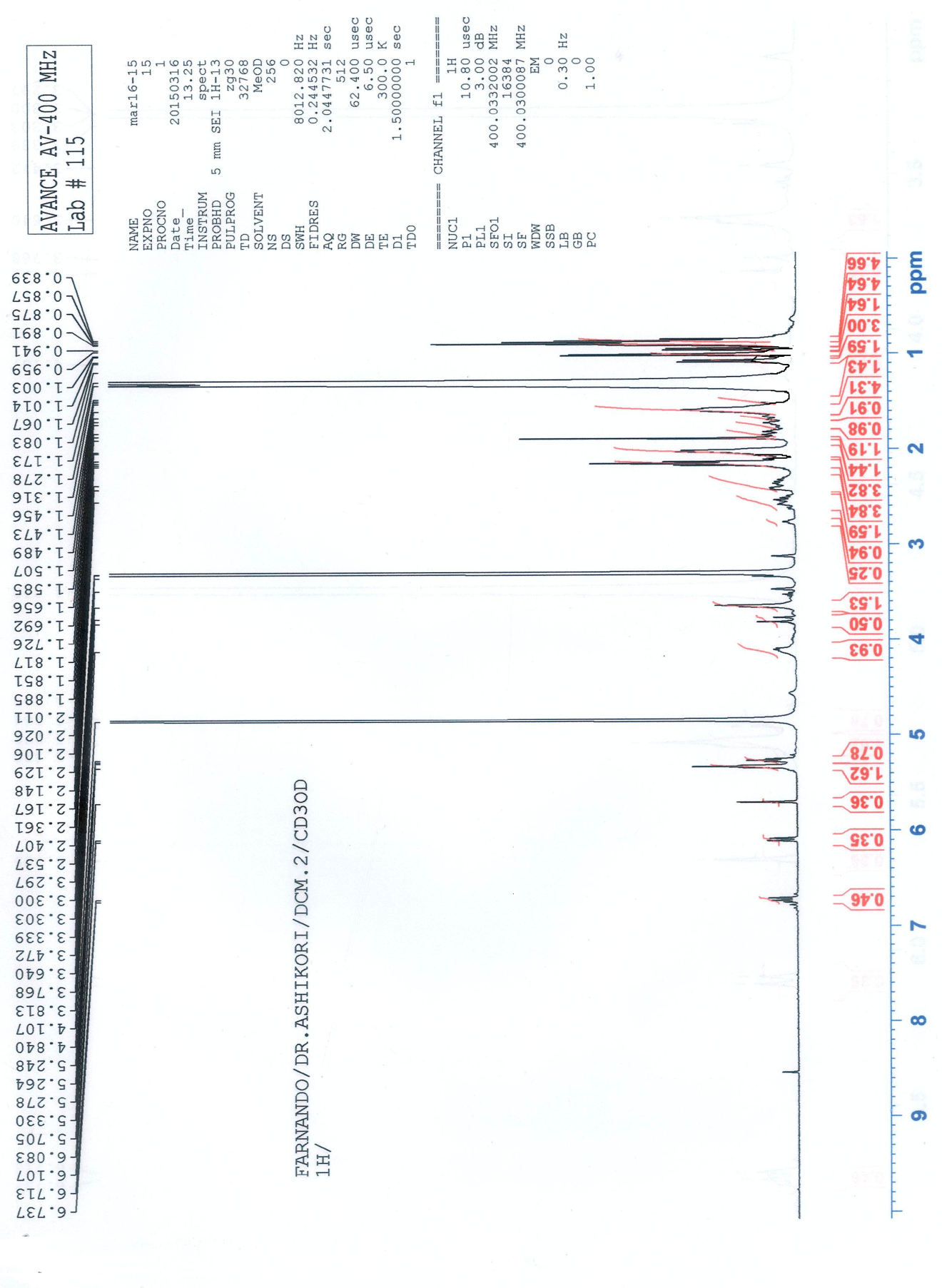
**

**Figure 1.** The ^1^H NMR spectrum (400 MHz) of Ergone, recorded in CD_3_OD.

**
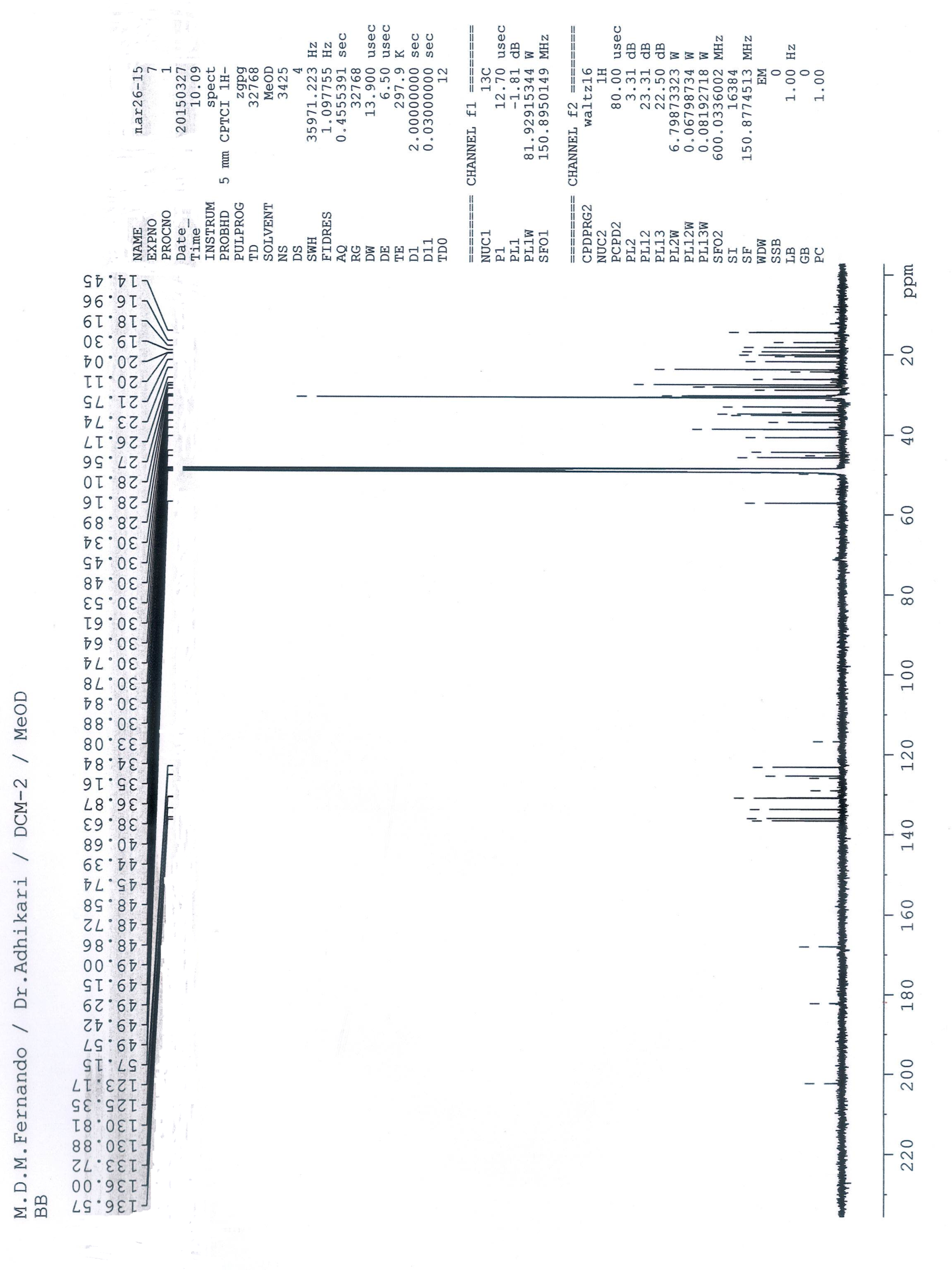
**

**Figure 2.** The ^13^C NMR spectrum (600 MHz) of ergone, recorded in CD_3_OD.

**
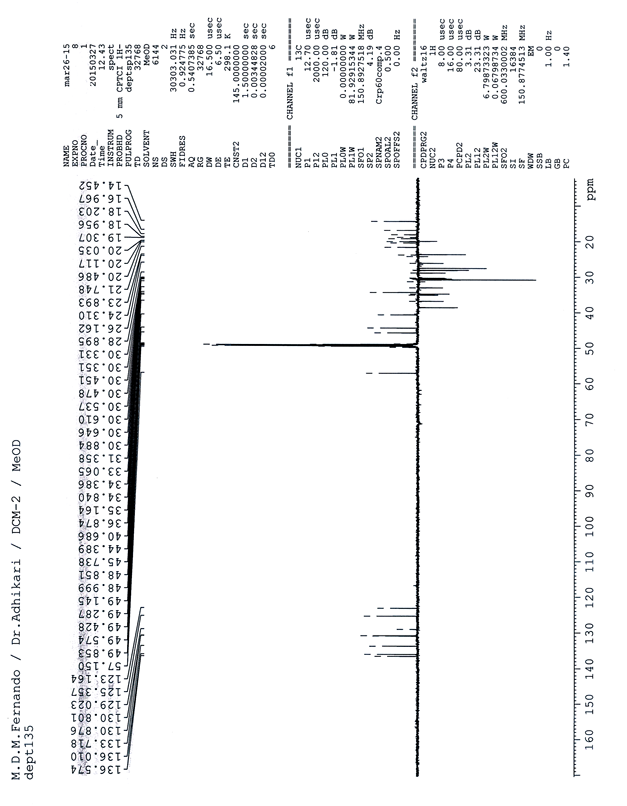
**

**Figure 3.** The DEPT135 NMR spectrum (600 MHz) of ergone, recorded in CD_3_OD.

**
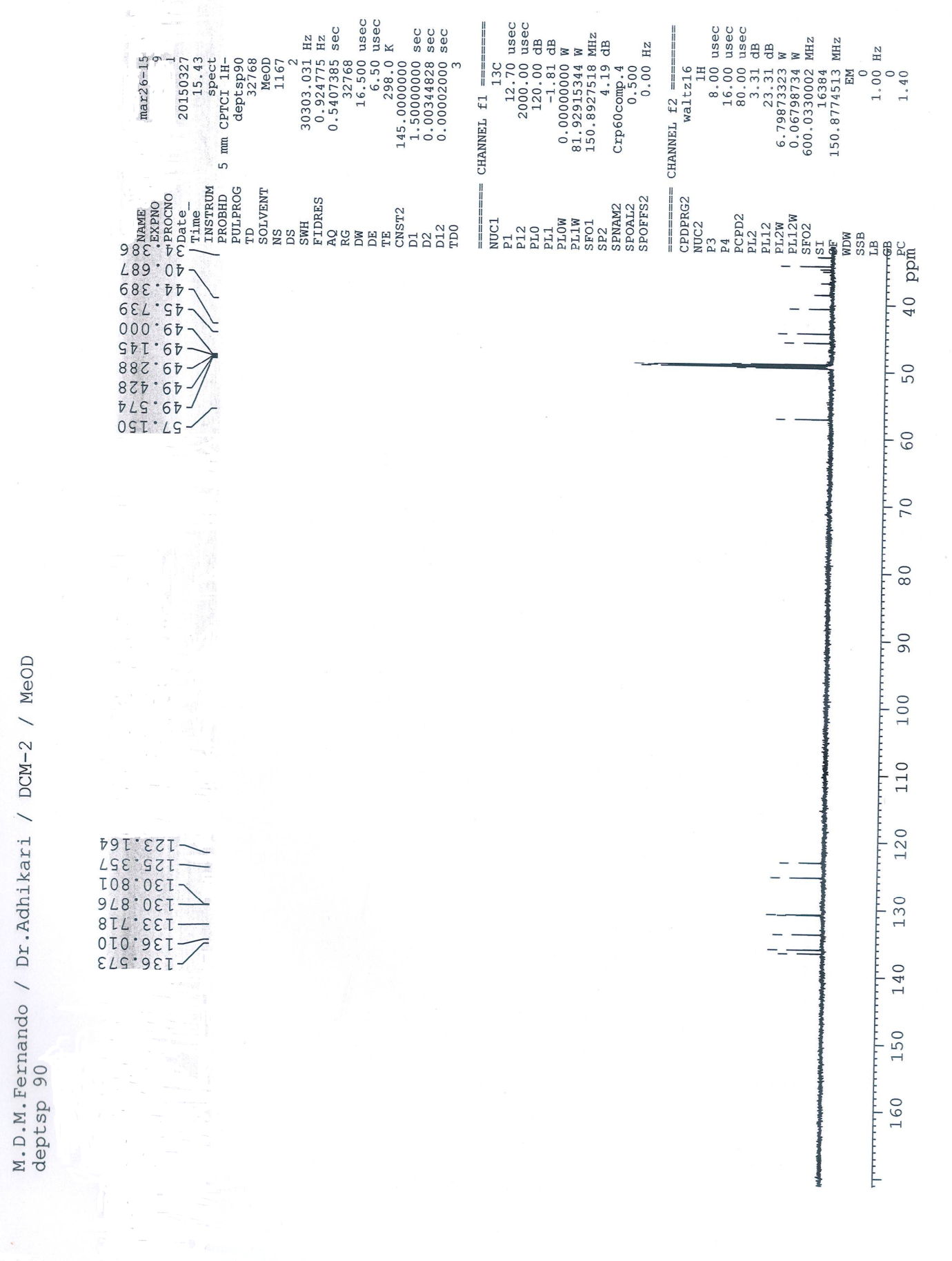
**

**Figure 4.** The DEPT90 NMR spectrum (600 MHz) of ergone, recorded in CD_3_OD.

**
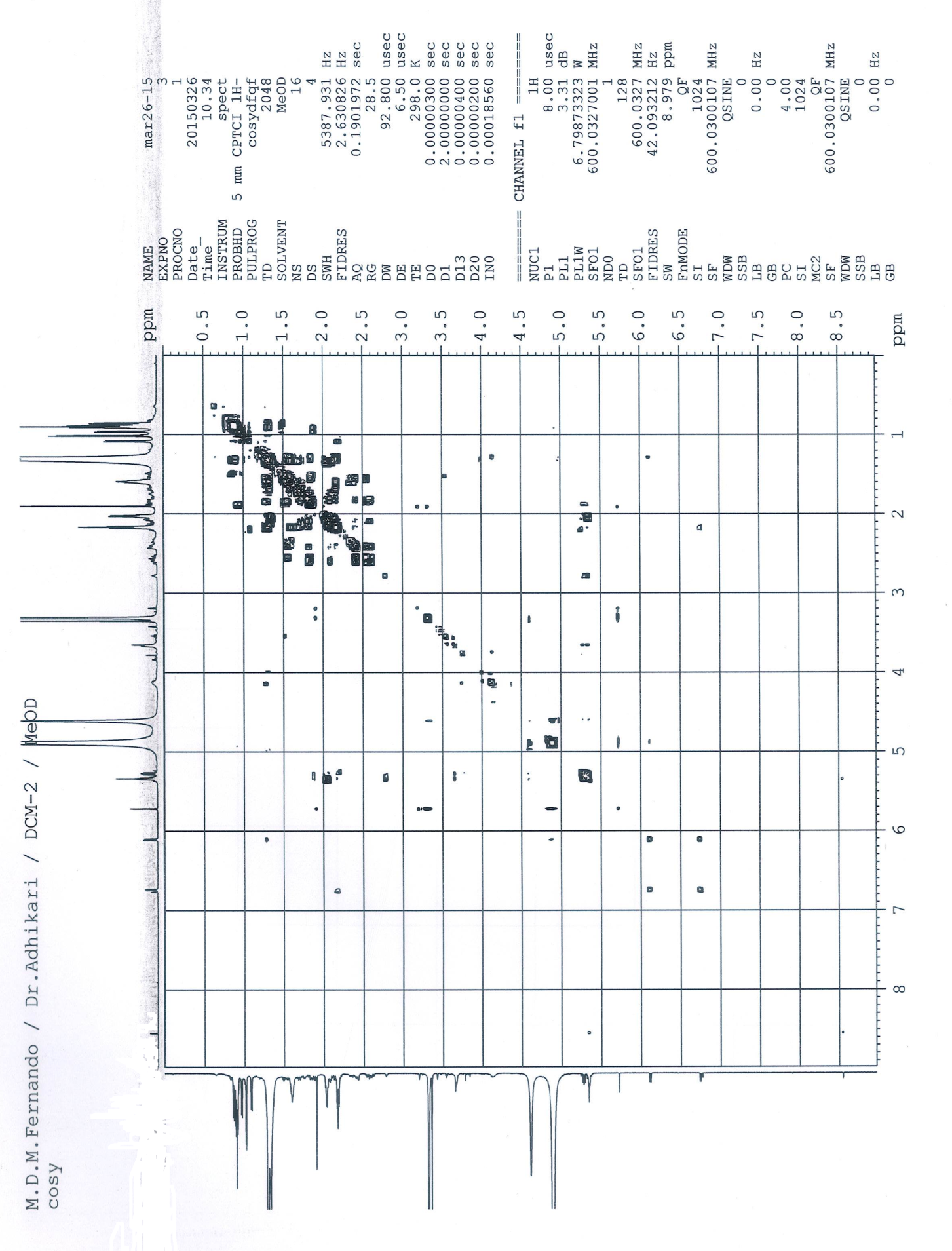
**

**Figure 5.** The COSY NMR spectrum (600 MHz) of ergone, recorded in CD_3_OD.

**
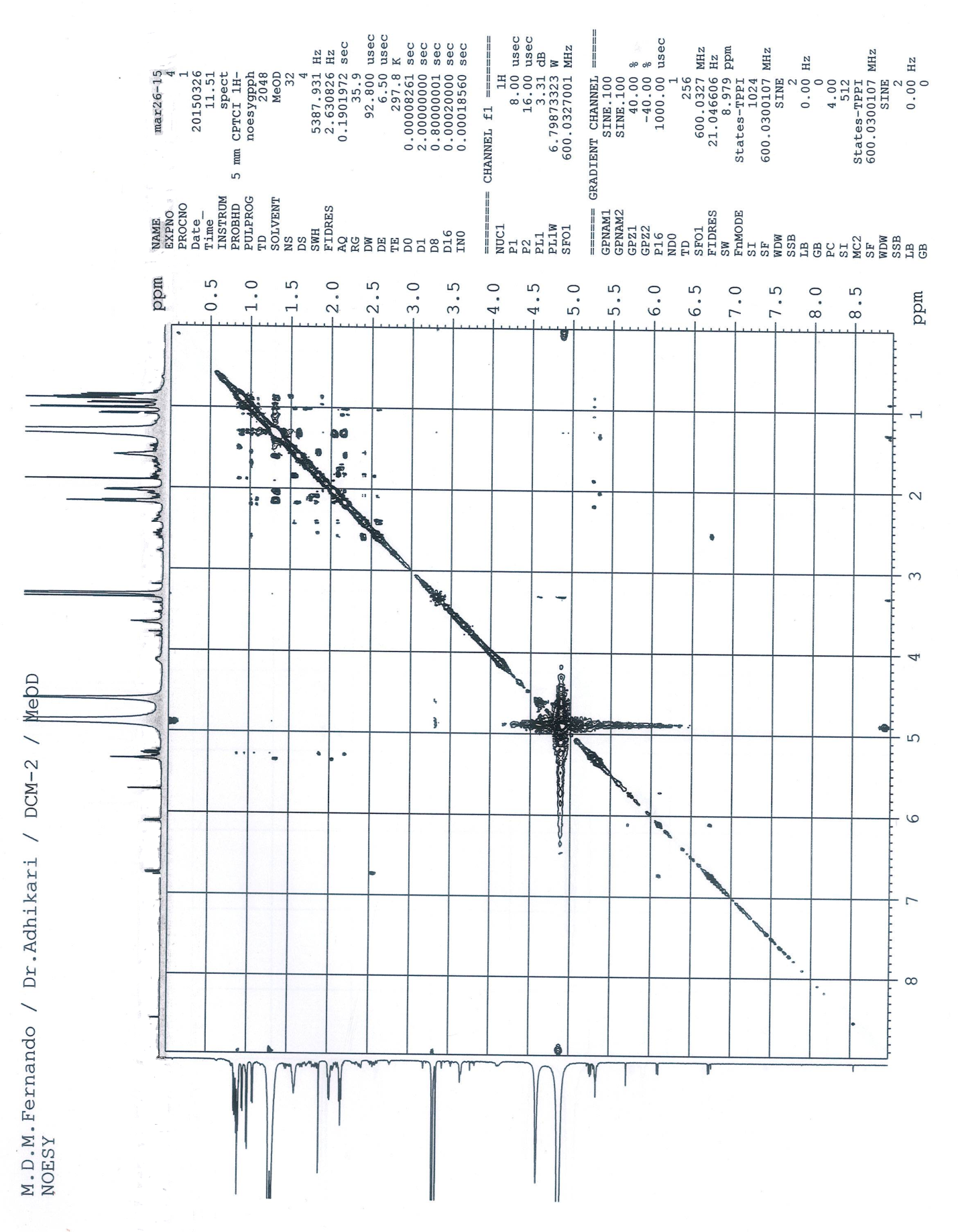
**

**Figure 6.** The NOESY NMR spectrum (600 MHz) of ergone, recorded in CD_3_OD.

**
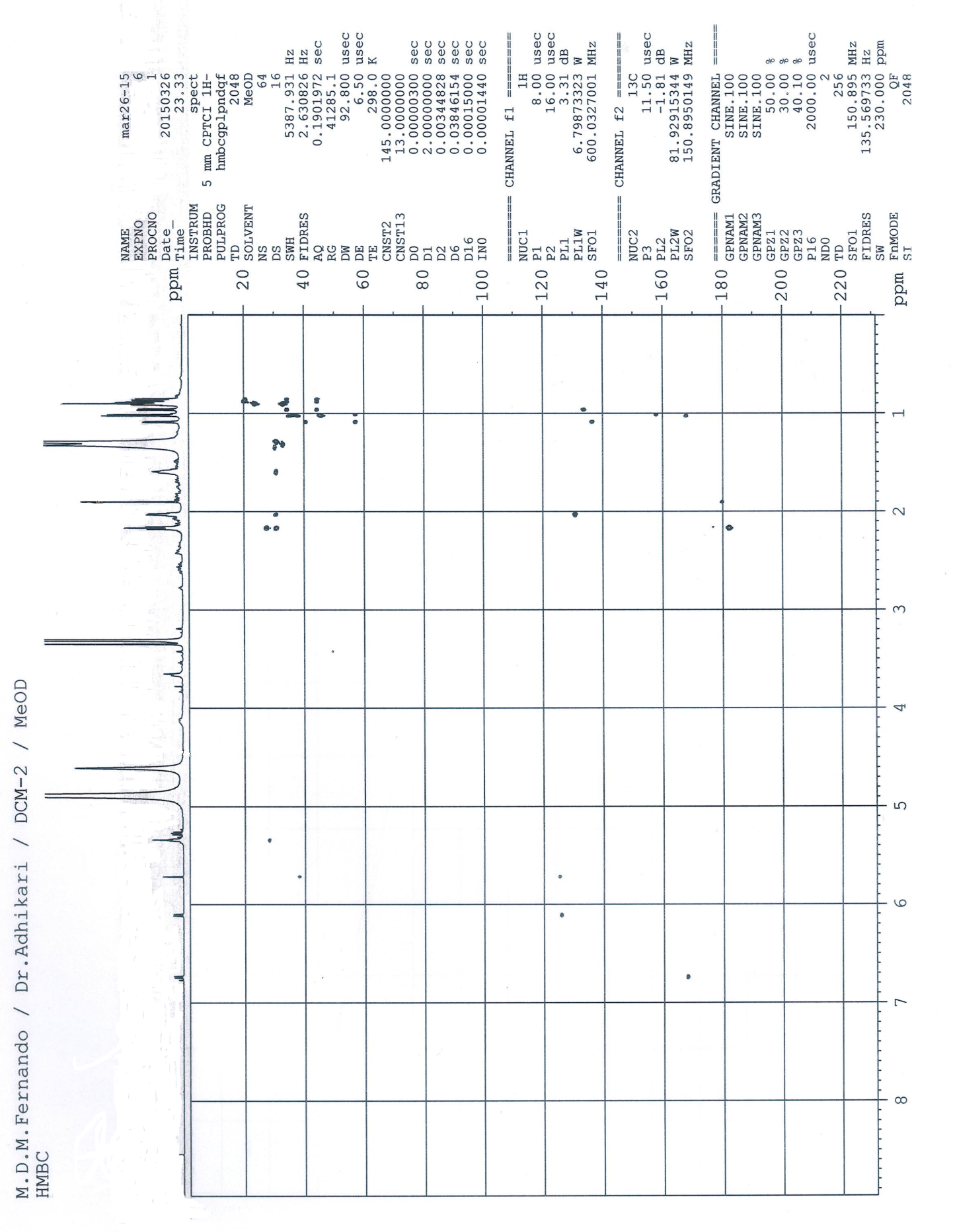
**

**Figure 7.** The HMBC NMR spectrum (600 MHz) of ergone, recorded in CD_3_OD.

**
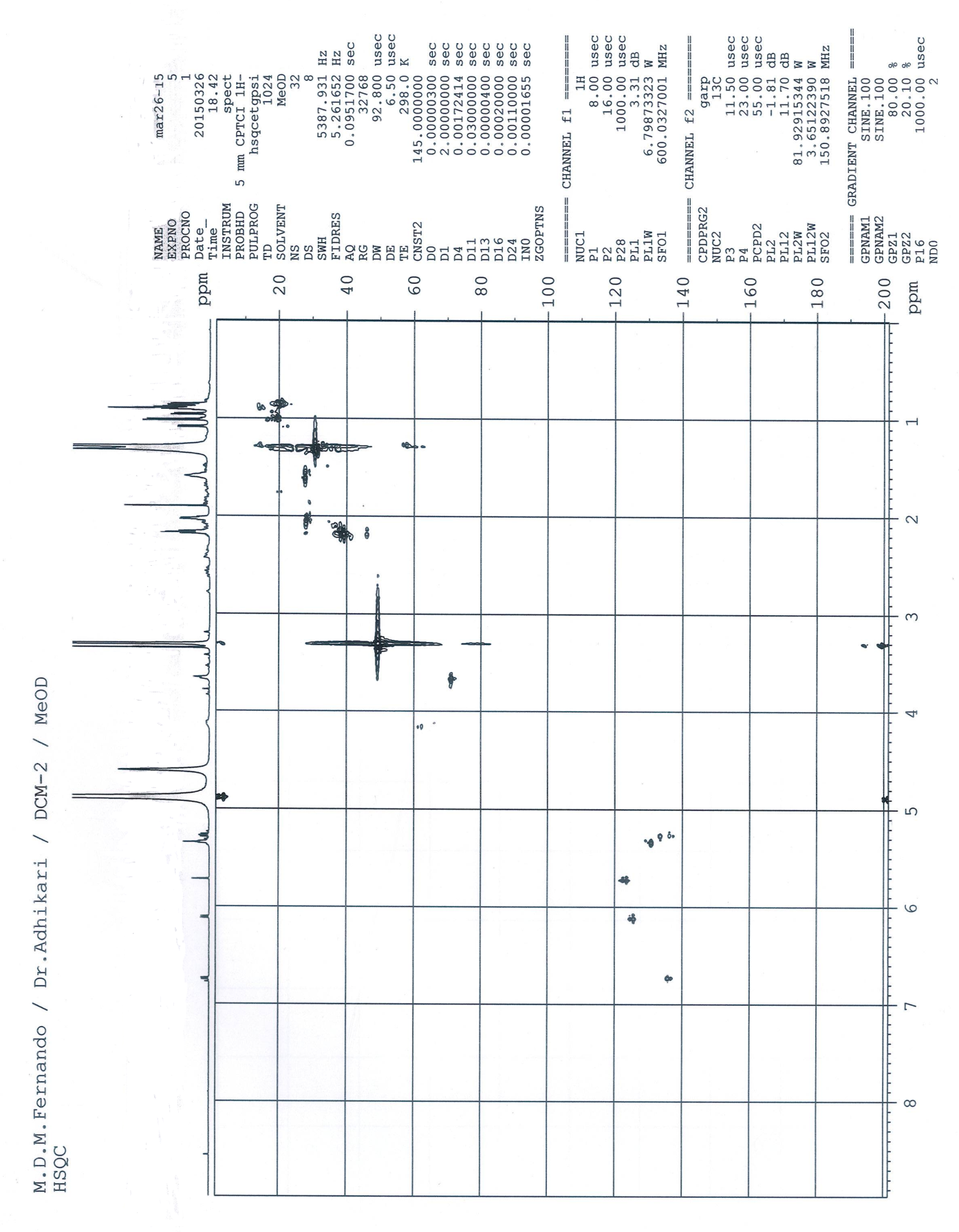
**

**Figure 8.** The HSQC NMR spectrum (600 MHz) of ergone, recorded in CD_3_OD.

**Figure 58.** The Mass spectrum of ergone, recorded in CD_3_OD.

**
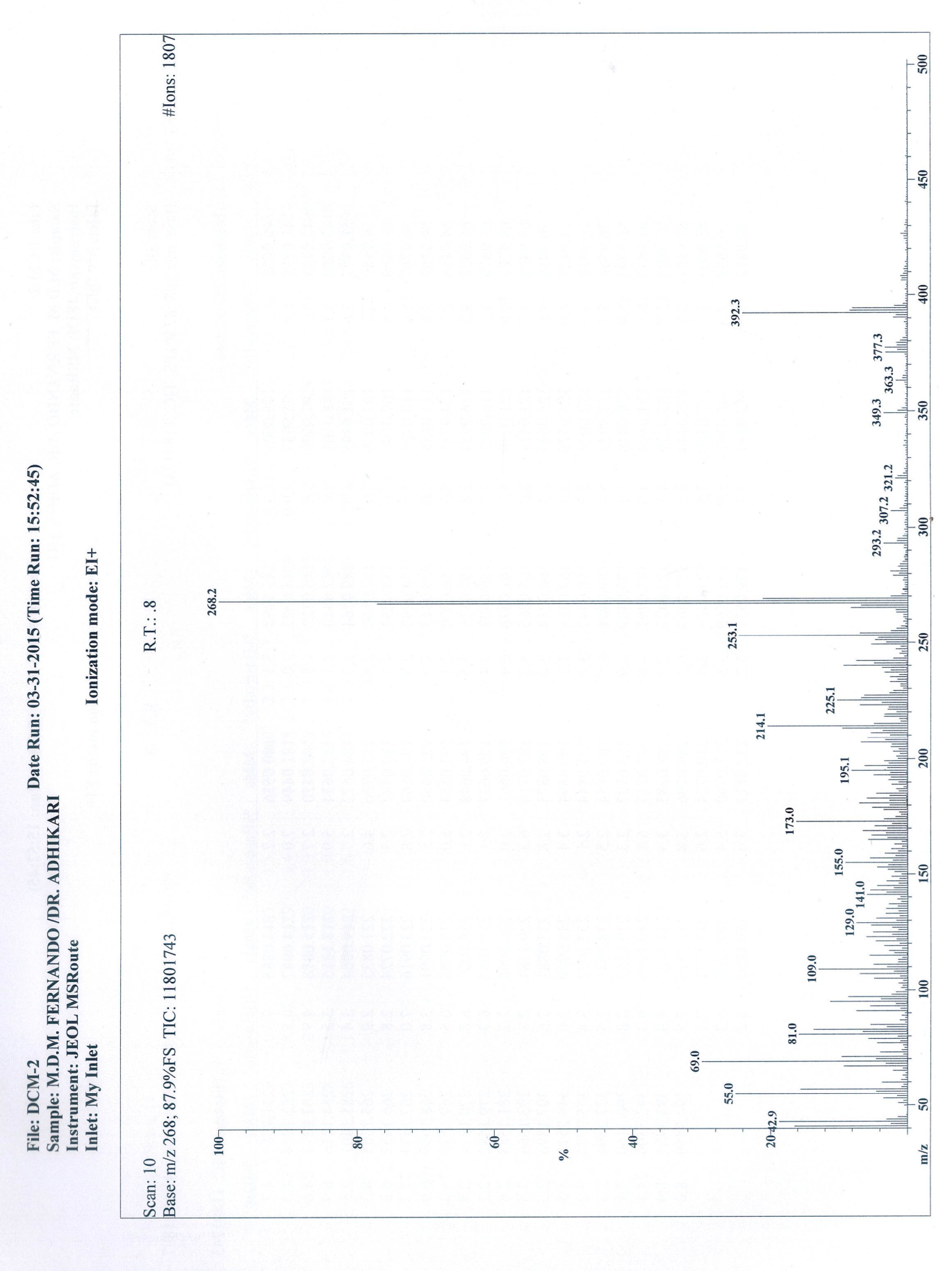
**

**Figure 9.** The Mass spectrum of ergone, recorded in CD_3_OD.
